# Dermal Delivery Enhancement of Natural Anti-Ageing Compounds from *Ocimum sanctum* Linn. Extract by Nanostructured Lipid Carriers

**DOI:** 10.3390/pharmaceutics12040309

**Published:** 2020-03-29

**Authors:** Wantida Chaiyana, Songyot Anuchapreeda, Suvimol Somwongin, Pachabadee Marsup, Kuan-Han Lee, Wei-Chao Lin, Shang-Chian Lue

**Affiliations:** 1 Department of Pharmaceutical Science, Faculty of Pharmacy, Chiang Mai University, Chiang Mai 50200, Thailand; suvimol_ampoo@hotmail.com (S.S.); pch.marsup@gmail.com (P.M.); 2 Research Center of Pharmaceutical Nanotechnology, Chiang Mai University, Chiang Mai 50200, Thailand; sanuchapreeda@gmail.com; 3 Division of Clinical Microscopy, Department of Medical Technology, Faculty of Associated Medical Sciences, Chiang Mai University, Chiang Mai 50200, Thailand; 4 Department of Pharmacy, Chia Nan University of Pharmacy and Science, Tainan 71710, Taiwan; lee.kuanhan@gmail.com; 5 Department of Cosmetic Science and Institute of Cosmetic Science, Chia Nan University of Pharmacy and Science, Tainan 71710, Taiwan; weilin@mail.cnu.edu.tw (W.-C.L.); myluemy@mail.cnu.edu.tw (S.-C.L.)

**Keywords:** *Ocimum sanctum* Linn., rosmarinic acid, anti-ageing, nanostructured lipid carriers, skin permeation

## Abstract

This study aimed to develop nanodelivery systems for enhancing the *Ocimum sanctum* Linn. extract delivery into the skin. Rosmarinic acid (RA) was used as a marker for the quantitative determination of the extract by high-performance liquid chromatography. Nanostructured lipid carriers (NLC), nanoemulsion, liposome, and niosome, were developed and characterized for internal droplet size, polydispersity index (PDI), and zeta potential using photon correlation spectroscopy. Irritation properties of each formulations were investigated by hen’s egg test on the chorioallantoic membrane. In vitro release, skin permeation, and skin retention are determined. NLC was suggested as the most suitable system since it enhances the dermal delivery of RA with the significant skin retention amount of 27.1 ± 1.8% (*p* < 0.05). Its internal droplet size, PDI, and zeta potential were 261.0 ± 5.3 nm, 0.216 ± 0.042, and −45.4 ± 2.4 mV, respectively. RA released from NLC with a sustained release pattern with the release amount of 1.29 ± 0.15% after 24 h. NLC induced no irritation and did not permeate through the skin. Therefore, NLC containing *O. sanctum* extract was an attractive dermal delivery system that was safe and enhanced dermal delivery of RA. It was suggested for further used as topical anti-ageing products.

## 1. Introduction

As the proportion of the ageing population continues to increase, the dermatological concerns of the aged grow in medical importance [1]. There are several ways to maintain the skin youthfulness, including iontophoresis, laser, derma roller, mesotherapy injection, botox injection, etc. On the other hand, the topical formulations are expansively used because of the lower price, ease of application, and lack of injury comparing to a medical device treatment. However, the effectiveness of those formulations is still suspicious as well as the effectiveness of the active ingredients.

Natural cosmetic active compounds are currently popular due to the growing concern and caution about the potential harmful effects of synthetic ingredients [2]. Our previous study revealed a potent anti-skin-ageing activity of *Ocimum sanctum* ethanolic extract, including antioxidation, anti-inflammation, inhibition against hyaluronic acid, and collagen fiber degradation inhibition [3]. Therefore, *O. sanctum* ethanolic extract is an attractive cosmetic active ingredient for anti-skin-ageing products. However, the extract would not be effective unless it could penetrate into viable epidermis or deeper into the dermis. Dermal delivery systems, especially nanodelivery systems, were hence important to overcome these problems.

There are various types of dermal nanodelivery systems, such as liposome, niosome, nanoemulsion (NE), nanostructured lipid carriers (NLC), etc. which are popular in the cosmetic market [4]. Liposome is effective in carrying several cosmetic active compounds since the products containing liposomes increase skin protection and beauty qualities [5]. Generally, liposomes are spherical, have uni- or multilamellar structures, and are a few nanometers in size. Liposomes have high efficiency in compound delivery to the dermal layer [6]. The major advantages of liposomes are their unique size, their ability to encapsulate both hydrophilic and hydrophobic bioactive compounds, the ability of UV-absorbing lipids to protect the skin, and the bioactive compounds that do not further degrade [6]. On the other hand, niosome is extensively used in cosmeceutical sectors since it has multiple advantages over liposomes such as deformable elastic vesicles that enhance the skin penetration, low toxicity, and higher protection of the bioactive compounds [7].

NE is submicron-sized emulsions which are under extensive investigation as drug and cosmetic carriers for improving the delivery of the active compounds [8]. The average droplet size of NE is usually between 100–500 nm. Due to its small droplet size, NE possesses stability against sedimentation or creaming with Ostwald ripening forming the main mechanism of NE breakdown [9]. NE is easily produced in large quantity by mixing a water-immiscible oil phase into an aqueous phase with a high stress, a mechanical extrusion process which is available worldwide [9].

NLC has been developed and used as an alternative carrier system to liposomes and emulsions since the 2000s [10]. The advantage of NLC is the higher loading capacity of active ingredients compared to solid lipid nanoparticles (SLN). Additionally, the firmer inclusion of the active ingredients inside the particle matrix would lead to better stability and longer shelf life [10]. NLC concentrate is more simply admixed to cosmetic formulations due to lower content in the formulations [11].

Therefore, the present study aimed to encapsulate *O. sanctum* ethanolic extract in various types of dermal nanodelivery systems, including NLCs, NEs, liposomes, and niosomes. Additionally, irritation properties, release profile, skin permeation, and skin retention of these dermal nanodelivery systems were investigated.

## 2. Materials and Methods

### 2.1. Plant Materials

The fresh plants of *O. sanctum* were purchased from a local market in Chiang Mai province, Thailand. After authentication, the herbarium specimen (number 023230) was deposited at the official Herbarium of the Faculty of Pharmacy, Chiang Mai University, Thailand. The *O. sanctum* dried powder was then prepared according to the method described by Chaiyana et al. [3].

### 2.2. Chemical Materials

Rosmarinic acid (RA), polysorbate 20 (Tween 20^®^), polysorbate 80 (Tween 80^®^), steareth-10 (Brij S10^®^), polyoxyethylene mono (octylphenyl) ether (Triton X-114^®^), decyl glucoside (Plantacare 2000^®^), polyethylene glycol 400 (PEG-400), propylene glycol, glycerin, cholesterol, stearic acid, glyceryl monostearate, cetyl palmitate, and bovine serum albumin (BSA) were purchased from Sigma-Aldrich (St. Louis, MO, USA). Tea seed (*Camellia oleifera* Abel.) oil, avocado (*Persea americana* Mill.) oil, and almond (*Prunus dulcis* Mill.) oil were cosmetic grade purchased from Namsiang (Chiang Mai, Thailand). HPLC grade acetonitrile was purchased from Merck (Darmstadt, Germany). Ethanol, ethyl acetate, *n*-hexane, and dimethyl sulfoxide (DMSO) were analytical grade purchased from Labscan (Dublin, Ireland).

### 2.3. Plant Extraction

The active compounds from *O. sanctum* were extracted using the method previously described by Chaiyana et al. [3]. Firstly, the nonpolar and semipolar compounds were removed by extracting with *n*-hexane and ethyl acetate, respectively. The residue of *O. sanctum* dried powder was then macerated in 95% ethanol for 24 h for 3 cycles with the assists of aluminum hot plate stirrer (Velp Scientific Inc., Milano, Italy) set at 50 °C. The pooled ethanol from 3 cycles of extraction was then filtered through No.1 Whatman filter paper (Merck KGaA, Darmstadt, Germany). The solvent was finally removed under vacuum using rotary evaporator (N-1001S-W, Eyela, Tokyo, Japan) until dryness at 50 °C. The obtained *O. sanctum* ethanolic extract was kept in light-resistance container in the refrigerator (4 °C) until further use.

### 2.4. High Performance Liquid Chromatography (HPLC)

HPLC analyses were performed using an HP1100 system with a UV detector set at 250 nm (Hewlett Packard, Palo Alto, CA, USA). A reversed phase column, (Eclipse XDB-C18 150 mm × 4.6 mm id, 5 μm Agilent, Palo Alto, CA, USA), was connected with an (Eclipse XDB-C18 guard column 4.0 mm × 4.6 mm id, 5 μm Agilent, Palo Alto, CA, USA) A gradient mobile phase system consisting of 0.5% formic acid (phase A) and acetonitrile (phase B) at a flow rate of 1.0 mL/min and 20 µL of 1 mg/mL *O. sanctum* ethanolic solution were injected. Each sample and mobile phase was filtrated through a 0.45-mm millipore filter, type GV (Millipore, Bedford, MA) prior to the HPLC injection. The gradient elution program was 90% A (0–2 min), 75% A (2–5 min) 0% A (5–8 min), and 90% A (8–10 min). All samples were analyzed in triplicate. RA was used as a marker for the quantitative analysis of *O. sanctum* ethanolic extract.

### 2.5. Nanodelivery Systems Development

#### 2.5.1. Nanostructured Lipid Carriers (NLCs)

NLCs were developed by using high-pressure homogenization [12]. The primary emulsion was premixed using high-performance dispersing instrument (IKA T 25 digital ULTRA-TURRAX^®^, Staufen, Germany) at 8000 rpm for 1 min and then passed though the high-pressure homogenizer (APV 1000, Wilmington, MA, USA) at 500 Pas for 5 cycles. Various factors on NLC development have been investigated, including solid lipid type, surfactant type, solid lipid amount, and liquid lipid amount. Tea seed oil was used as liquid lipid in the NLCs development, whereas different types of solid lipid were used, including stearic acid, glyceryl monostearate, and cetyl palmitate. Various types of surfactants were used in the present study, including Tween 20^®^, Tween 80^®^, and Plantacare 2000^®^. Various formulations of NLCs were developed as shown in Table 1.

#### 2.5.2. Liposome and Niosome

Liposome and niosome were prepared by the Bangham method or thin-film hydration method [13]. Various factors affecting liposome and niosome formation, including lecithin, surfactant, and cholesterol amount, were investigated. Six formulations of liposome and niosome as shown in Table 2 were developed. The hydrophobic excipients, including lecithin and cholesterol, were dissolved in ethanol and transferred into a round bottom flask. The flask was then connected to a rotary evaporator (N-1001S-W, Eyela, Tokyo, Japan), and a water bath (OSB-2000, Eyela, Tokyo, Japan) was set at the temperature of 40 °C. The vacuum was applied to the flask to evaporate the solvent, and a homogeneous lipid film was formed on the flask wall. The dry lipid film was kept overnight to remove the traces of ethanol. The lipid film was then hydrated by the addition of DI water, and the flask was rotated around 30 min until the lipid film was completely hydrated. The liposome and niosome dispersion were then produced by hand shaking for 10 min. The formulations were then kept at room temperature in tight containers until further investigations.

#### 2.5.3. Nanoemulsion

NEs were prepared by high-pressure homogenization [14]. Briefly, the primary emulsion was premixed using high-performance dispersing instrument (IKA T 25 digital ULTRA-TURRAX^®^, Staufen, Germany) at 8000 rpm for 1 min and then passed though the high-pressure homogenizer (APV 1000, Wilmington, MA, USA) at 500 Pas for 5 cycles. Various factors affecting the NE formation were investigated, including oil type, oil amount, surfactant type, and humectant type. Various oil types, including tea seed oil, avocado oil, and almond oil, were used for NE development. Additionally, the effect of different surfactant types, including Tween 20^®^, Brij S10^®^, and Triton X-114^®^ as well as different humectant types, including glycerin, propylene glycol, and PEG-400 were also investigated. Various formulations of NE as shown in Table 3 were developed. The formulations were kept at room temperature in tight containers until further used.

#### 2.5.4. Characterization of Nanodelivery Systems

##### Particle Size, Size Distribution, and Zeta Potential Measurement

The formulations were diluted 1000 times with DI water before the particle size, size distribution, and zeta potential analysis were carried out using photon correlation spectroscopy (Zetasizer^®^ version 5.00, Malvern Instruments Ltd., Malvern, UK). The sizing measurements were carried out at a fixed angle of 173°. The reported results were mean and S.D. of at least ten measurements on each sample.

##### Stability Study

Each nano-formulation was subjected to eight heating–cooling cycles (24 h each at 4 °C and 45 °C) and then characterized by means of external appearance, particle size, size distribution, and zeta potential measurement as previous described.

### 2.6. Development of Nanodelivery Systems Containing O. sanctum Ethanolic Extract

*O. sanctum* ethanolic extract was incorporated into the nanodelivery systems which had a good external appearance, small internal droplet size, narrow PDI, and pronounced zeta potential value and was stable after the stability test. The concentration of *O. sanctum* ethanolic extract in the nanodelivery systems was 0.1% *w/w*, which was 10 times the effective concentration in the matrix metalloproteinase-1 (MMP-1) inhibitory activity reported in the previous study [4]. In the procedure of NLC and NE development, *O. sanctum* ethanolic extract was pre-dissolved in Plantacare 2000^®^ and Tween 20^®^, respectively, before mixing with other components in further primary emulsion preparation processes. After that, the primary emulsions containing *O. sanctum* ethanolic extract were passed though the high-pressure homogenizer (APV 1000, Wilmington, MA, USA) at 500 Pas for 5 cycles. The entrapment efficiency of NLCs and NEs investigated by indirect method were 87.4 ± 5.6% *w/w* and 94.8 ± 6.7% *w/w*, respectively.

### 2.7. Irritation Study by Hen’s Egg Test on the Chorioallantoic Membrane (HET-CAM)

The irritation of 0.1% *w/w O. sanctum* ethanolic extract solution, blank nanodelivery systems, and nanodelivery systems containing 0.1% *w/w O. sanctum* ethanolic extract was investigated using hen’s egg test on the chorioallantoic membrane (HET-CAM) with slight modifications [15,16,17]. This experiment was convenient and famous because the ethical approval did not need to be applied when the age of animal’s embryo was less than half of the total incubation period [18]. The hen eggs obtained after the fertilization were incubated for 7 days in the hatching chamber at the temperature of 37.5 ± 0.5 °C and humidity of 55 ± 7%. The CAM preparation was performed by flooding the eggs with light to indicate the air chamber, by opening the eggshell, and by removing the white egg membrane. The CAM was immediately exposed to 200 µL of the samples. The specific alterations of each samples on the membrane and blood vessel network were examined as haemorrhage, lysis, and coagulation. The haemorrhage was observed as the bleeding out from blood vessels of the vascularized CAM. The lysis was indicated by a disappearance of small blood vessels on the CAM as a consequence either of bleeding, dystonia of these fine vessels, or real disintegration. The coagulation included either intravascular coagulation (thrombosis) or extravascular coagulation of proteins on the CAM that normally increases the CAM opacity. The time of first occurrence of the three abovementioned endpoints was registered within a maximum time period of 5 min. From these data, an irritation index (RI) was calculated using the following equation:

(1) IS =((301−t(h)×5)/300((301−t(l))×7)/300((301−t(c))×9)/300,

where *t(h)* is the time of first vascular haemorrhage detected, *t(l)* is the time of first vascular lysis detected, and *t(c)* is the time of first vascular coagulation detected. Irritation classification was based on IS as 0.0−0.9, non-irritation; 1.0−4.9, slight irritation; 5.0−8.9, moderate irritation; and 9.0−21.0, severe irritation. The experiments were done in duplicates.

### 2.8. In Vitro Release Study

The in vitro release study of *O. sanctum* extract was investigated using a slightly modified method of Chaiyana, et al. [19]. Briefly, 1.0 mL of each nanodelivery systems containing 0.1% *w/w O. sanctum* extract was placed into 2.5 × 2.5 cm dialysis bags (Cellu Sep T2, Membrane Filtration Products Inc., Frilabo, Maia, Portugal). Each dialysis bag was then introduced into a media, composed of 4% *w/w* BSA and 10% *w/w* Tween 80^®^ in Phosphate-buffered saline (PBS), pH 5.5. The media was continuously stirred by a hot plate stirrer (Velp Scientific Inc., Milano, Italy) set at 32 °C. The medium was removed at specific time intervals of 1, 2, 4, 8, and 24 h. Once the medium was removed, the same amount of fresh medium was immediately replaced. The amount of the released *O. sanctum* extract was determined using HPLC as described previously. All the experiments were done in triplicate.

### 2.9. In Vitro Skin Permeation and Skin Retention Study

#### 2.9.1. Skin Preparation

Full-thickness skin from the flank area of stillborn piglets which accidentally died before birth was used for the in vitro skin permeation studies [20,21]. Dead stillborn piglets in placenta were obtained from a local farm in Chiang Mai province, Thailand. After the placenta removal, the stillborn piglets were washed with tap water and the hair was trimmed off using a hair clipper. The skin pieces from the flank area were carefully dissected with a surgical blade. After washing in PBS, pH 5.5, the skin was wrapped in tin foil and stored at −20 °C for up to 1 month [22]. Prior to the permeability studies, the skin was defrosted and hydrated in PBS, pH 5.5 overnight at room temperature. The subcutaneous fat layer was carefully trimmed off by using surgical scissors before placing the skin onto the vertical Franz diffusion cells.

#### 2.9.2. Skin Permeability

Vertical Franz diffusion cells was used for the in vitro skin permeation studies [19]. Briefly, 13 mL of receptor media, composed of PBS, pH 7.4 thermostated at 37 °C, was stirred constantly with a hot plate stirrer (Velp Scientific Inc., Milano, Italy) set at 100 rpm. The skin was mounted on Franz diffusion cells for 1 h prior to the experiment to ensure the skin equilibration. The direct current was measured along the donor and receptor compartments of the diffusion cell both filled with PBS, pH 7.4 using handheld ohmmeter (Hotek Technologies, Inc, Tacoma, WA, USA). Only skin samples having a resistance higher than 10 kΩ/cm were used for skin permeability measurements [23].

To investigate skin permeability, 500 µL of the samples was applied onto the skin surface. The blank formulations without *O. sanctum* extract were used as a control. Aliquots of 500 µL of the receptor media were withdrawn at predetermined time intervals of 1, 2, 4, 8, and 24 h and immediately replaced with an equal amount of fresh media (PBS, pH 7.4) to maintain a sink condition. The amount of permeated *O. sanctum* extract was determined using HPLC as described previously. All formulations were tested in triplicate, using skin samples of different stillborn piglets.

#### 2.9.3. Skin Retention

After 24 h of the sample application, the amount of *O. sanctum* extract retained in the skin layer were investigated. Briefly, the skins obtained from the permeation study were removed from the Franz diffusion cell and rinsed with DI water. The skin was then homogenized in 1:1 ethanol and PBS, pH 7.4 using high-performance dispersing instrument (IKA T 25 digital ULTRA-TURRAX^®^, Staufen, Germany) and stored in a refrigerator for at least 12 h to allow complete extraction. The amount of the retained *O. sanctum* extract was determined using HPLC as described previously. All formulations were tested in triplicate, using skin samples from different animals.

### 2.10. Statistical Analysis

All data were presented as a mean ± standard deviation (S.D.). Statistical significance was assessed by the one-way analysis of variance (ANOVA) followed by post hoc tests using SPSS 17.0 for Windows (SPSS Inc., Chicago, IL, USA). The probability values of * *p* < 0.05, ** *p* < 0.01, and *** *p* < 0.001 were considered significant.

## 3. Results

### 3.1. O. sanctum Extract

**Figure 1:**
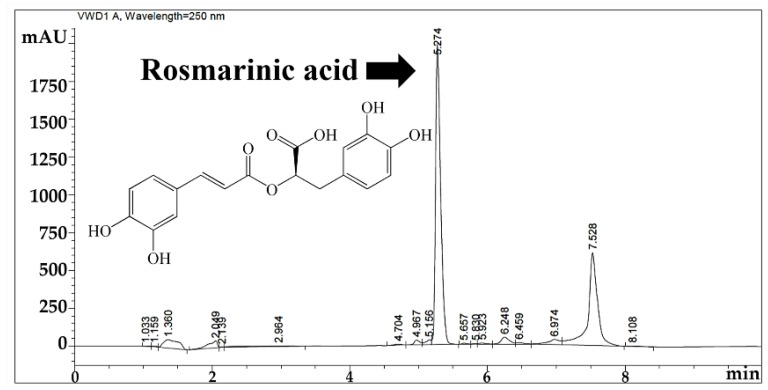
HPLC chromatogram of *O. sanctum* ethanolic extract: Rosmarinic acid (RA) was detected as a major component.

*O. sanctum* ethanolic extract was a greenish semisolid mass with the yield of 6.53% *w/w*. HPLC chromatogram of the extract is shown in Figure 1. RA was noted as a major component of *O. sanctum* ethanolic extract since it was detected as the predominant peak defined by the largest area under the curve (AUC) at the retention time of 5.274 min. The results were correlated well with our previous study that reported that 19.3% *w/w* of the ethanolic extract of *O. sanctum* was RA [3]. Additionally, RA has been previously reported as a component in the ethanolic extract and volatile oil of *O. sanctum* [24,25]. Therefore, RA was used as a marker for the quantitative determinations of *O. sanctum* ethanolic extract in further release, skin permeation, and skin retention study.

### 3.2. Nanodelivery Systems

#### 3.2.1. Nanostructured Lipid Carriers (NLCs)

**Figure 2:**
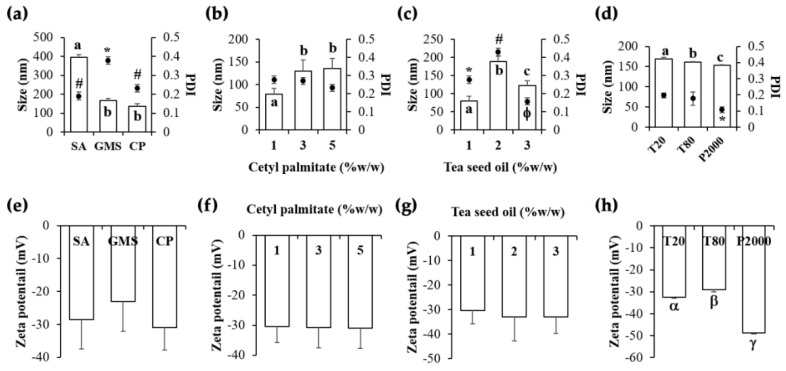
Particle size and polydispersity index (PDI) of nanostructured lipid carriers were developed using (**a**) different types of solid lipid at the same concentration of 5% *w/w*, (**b**) various amounts of cetyl palmitate, (**c**) various amounts of tea seed oil, and (**d**) different types of surfactants at the same concentration of 2.5% *w/w*. Zeta potential of nanostructured lipid carriers were developed using (**e**) different types of solid lipid, (**f**) various amounts of cetyl palmitate, (**g**) various amounts of tea seed oil, and (**h**) different types of surfactants. Different types of solid lipids included stearic acid (SA), glyceryl monostearate (GMS), and cetyl palmitate (CP). Different types of surfactants included Tween 20^®^ (T20), Tween 80^®^ (T80), and Plantacare 2000^®^ (P2000). The letters, a, b, and c denote values that were significantly different in particle size (*p* < 0.05), whereas, the symbols, *, #, and φ denote values that were significantly different in PDI (*p* < 0.05). The symbols, α, β, and γ denote values that were significantly different in zeta potential (*p* < 0.05).

Effects of various factors on the NLCs characteristics are shown in Figure 2, including solid lipid type, solid lipid amount, liquid lipid amount, and surfactant type. It was remarked that all factors affected the particle size and PDI of the NLCs whereas only the surfactant type affected the zeta potential values.

Cetyl palmitate was suggested as the most appropriate solid lipid for NLCs development since it could generate NLCs with smaller particle size, narrower PDI, and the least zeta potential value (Figure 2a,e). Although the particle size of NLCs containing cetyl palmitate and glyceryl monostearate were not significantly different (*p* > 0.05), the PDI of NLCs containing cetyl palmitate was significantly narrower than that of glyceryl monostearate (*p* < 0.05). It was likely that the internal droplet size and PDI tended to relate with the melting point of a solid lipid phase. A solid lipid with lower melting point tended to yield NLCs with smaller internal droplet size. Cetyl palmitate could be used to generate NLCs with the smallest particle size due to its lowest melting point (54 °C) comparing to glyceryl monostearate (57–65 °C) and stearic acid (69.3 °C). The reason was because cetyl palmitate took a longer duration before it turned into the solid state, which led its molten droplet to pass through an orifice of the high-pressure homogenizer and to be torn apart into smaller sizes with more uniform size distribution via a turbulent and cavitation theory [26]. The PDI of 0.232 ± 0.02 of this formulation indicated a relatively narrow size distribution of the NLC particles. Furthermore, NLCs containing cetyl palmitate has the least zeta potential value, which could prevent the aggregation of NLC particles that would lead to a more stability. Therefore, cetyl palmitate was selected, and the suitable amount of cetyl palmitate in the NLC formulation was investigated.

The higher amount of cetyl palmitate could produce NLCs with larger particle size, whereas no effect on PDI and zeta potential value was observed (Figure 2b,f). These results related well with the previous study about cetyl palmitate-based NLCs which noted the higher mean particle sizes were obtained in the higher concentration of the lipid phase [27]. Additionally, double amounts of the active compound (coenzyme Q10) could be completely encapsulated in the NLCs with higher content of cetyl palmitate [27]. Therefore, the highest amount of cetyl palmitate in the present study, which was 5% *w/w*, was selected for NLC development since it might lead to higher entrapment efficiency.

Liquid lipid is another component which differentiates NLCs from SLNs by making the imperfection structure which led to various advantages over SLNs, including higher loading capacity, better stability, and longer shelf life [12]. In the present study, the concentration of liquid lipid (tea seed oil) in NLC formulations affected their particle size and PDI, whereas no effect on the zeta potential value was detected (Figure 2c,g). Although lower amounts of tea seed oil could produce the NLCs with smaller particle size, the significantly narrowest PDI was found in the formulation with 3% *w/w* of tea seed oil (*p* < 0.05). Since liquid lipid in the NLCs provided a kind of crystalline imperfection [28], its concentration altered the NLC characteristics. The results were in a good agreement with previous studies since there was no tendency of internal droplet size or PDI with the increasing concentration of liquid lipid amount whereas an optimum liquid lipid amount was obviously observed [28,29]. Therefore, 3% *w/w* of tea seed oil, which was an optimal concentration that yielded NLC with small particle size, the significantly narrowest PDI, and lower zeta potential value, would be the most appropriate amount for the NLCs preparation.

Interestingly, surfactant type was the major factor affecting the characteristics of NLCs since it affected all parameters, including particle size, PDI, and zeta potential (Figure 2d,h). Plantacare 2000^®^ was the most suitable surfactant for the NLCs development since it could produce the NLCs with the significantly smallest particle (152.5 ± 1.4 nm), narrowest PDI (0.108 ± 0.017), and most pronounced zeta potential value (−48.8 ± 0.4). The results were in a good accordance with the previous study that reported that Plantacare 2000^®^ and its combination with other surfactants could produce the best NLCs [30]. In conclusion, the most suitable NLC formulation was A9 which was composed of 5% *w/w* cetyl palmitate, 3% *w/w* tea seed oil, 2.5% *w/w* Plantacare 2000^®^, and 91.5% *w/w* DI water.

#### 3.2.2. Liposome and Niosome

**Figure 3:**
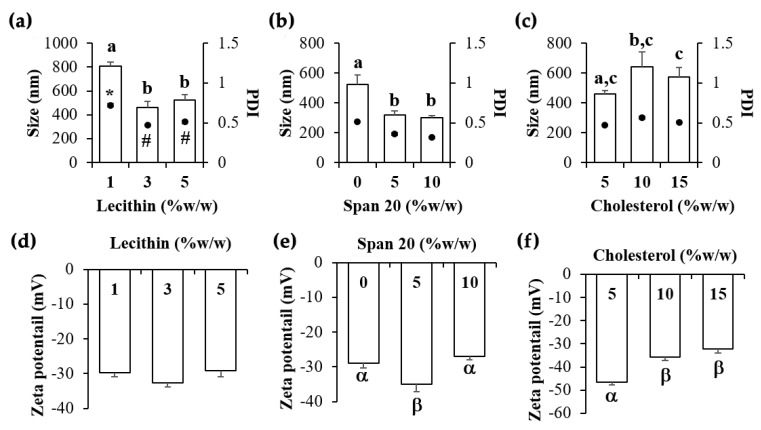
Particle size and polydispersity index (PDI) of liposome and niosome were developed using (**a**) various amount of lecithin, (**b**) Span 20^®^, and (**c**) cholesterol. Zeta potential of liposome and niosome were developed using (**d**) various amount of lecithin, (**e**) Span 20^®^, and (**f**) cholesterol. The letters, a, b, and c denote values that were significantly different in particle size (*p* < 0.05), whereas the symbols * and # denote values that were significantly different in PDI (*p* < 0.05). The symbols α and β denote significantly different values in zeta potential (*p* < 0.05).

Effects of lecithin amount on liposome are shown in Figure 3. Higher amounts of lecithin could produce liposomes with smaller particle size and narrower PDI (Figure 3a), whereas no effect on the zeta potential value was detected. The likely explanation was because smaller liposomal particles required larger amount of phosphatidylcholine, a major component of lecithin, to form a lipid bilayer wall. The results were related well with a previous study that reported that increasing the amount of marine lecithin from 1% to 10% led to smaller size of liposome [31]. Additionally, higher entrapment efficiency increased with higher lecithin content [31,32]. Therefore, 3% *w/w* of lecithin was selected for further study since it could produce a liposome with small particle size (464.0 ± 50.1 nm), narrowest PDI (0.470 ± 0.013), and most pronounced zeta potential value (−32.6 ± 1.3). In this formulation, 1% *w/w* of cholesterol was used as a stabilizer since cholesterol plays a strategic role in liposome composition [33]. Therefore, B2 was selected as the most suitable liposome for further incorporation of *O. sanctum* extract.

In the aspect of niosome development, nonionic surfactant was used instead of lecithin [34]. The effects of Span 20^®^ and cholesterol amount on the characteristics of niosome are shown in Figure 3. The presence of nonionic surfactant led to smaller internal droplet size and narrower PDI of the niosome (Figure 3b) due to the interfacial tension reduction between niosomal particles and dispersion medium. However, higher amounts of Span 20^®^ significantly affected neither internal droplet size nor PDI. Therefore, the suggested amount of Span 20^®^ was 5% *w/w* since it could produce the niosome with the most pronounced zeta potential value (Figure 3e). On the other hand, higher amount of cholesterol led to significantly larger internal droplet size and less pronounced zeta potential value (*p* < 0.05) (Figure 3c,f). The likely explanation might be due to cholesterol increasing the chain order of liquid-state bilayers and higher content of cholesterol leading to the increase of hydrodynamic diameter [35]. Therefore, only 5% *w/w* of cholesterol was enough to stabilize the niosome. In brief, the most suitable niosome (formulation B6), produced from 5% *w/w* Span 20^®^, 5% *w/w* of cholesterol, and 90% *w/w* DI water, had the smallest particles (460.0 ± 20.9 nm), the narrowest PDI value (0.472 ± 0.002), and the most pronounced zeta potential value (−46.4 ± 1.2).

#### 3.2.3. Nanoemulsion

**Figure 4:**
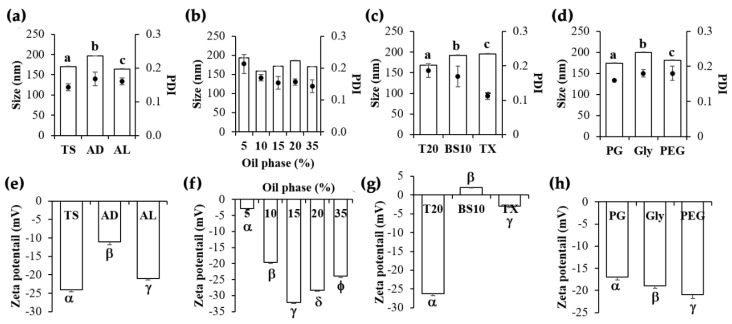
Particle size and polydispersity index (PDI) of nanoemulsion were developed using (**a**) different types of oils at the same concentration of 35% *w/w*, (**b**) various amount of oil phase, (**c**) different types of surfcatants at the same concentration of 7.5% *w/w*, and (**d**) different types of humectants at the same concentration of 7.5% *w/w*. Zeta potential of nanoemulsion were developed using (**e**) different types of oils, (**f**) various amount of oil phase, (**g**) different types of surfcatants, and (**h**) different types of humectants. Different types of oils included tea seed oil (TS), avocado oil (AD), and almond oil (AL). Different types of surfactants included Tween 20^®^ (T20), Brij S10^®^ (BS10), and Triton X-114^®^ (TX). Different types of humectant included propylene glycol (PG), glycerin (Gly), and polyethylene glycol 400 (PEG). The letters, a, b, and c denote values that were significantly different in particle size (*p* < 0.05), whereas the symbols, α, β, γ, δ, and ϕ denoted values that were significantly different in zeta potential (*p* < 0.05).

Various factors affecting NE development have been investigated, including oil type, oil phase amount, surfactant type, and humectant type. The results remarked that all factors affected the NE characteristics as shown in Figure 4. Tea seed oil was suggested as the most suitable oil phase since it could produce NE with small internal droplet size, the narrowest PDI, and the most significantly pronounced zeta potential value (*p* < 0.05) (Figure 4a,e). However, different amounts of tea seed oil had no effect on the internal droplet size and PDI (Figure 4b). Since tea seed oil was mainly composed of oleic acid (C18:1), linoleic acid (C18:2), and palmitic acid (16:0) [36], of which the pKa value was 4.0 [37], these fatty acids were hence in an ionized form and possessed more pronounced zeta potential value at higher pH of the NE system (pH = 5.92 ± 0.08). Consequently, higher tea seed oil content led to more pronounced zeta potential values (Figure 4f), but the maximum pronounced zeta potential was detected at the tea seed oil content of 15% *w/w* and zeta potential was less pronounced at higher oil content over 15% *w/w*. Therefore, 15% *w/w* tea seed oil was suggested for NE preparation.

Tween 20^®^ was selected as a surfactant in the system since it produced an NE with the significantly smallest internal droplet size (*p* < 0.05), narrow PDI, and the significantly most pronounced zeta potential value (*p* < 0.05) (Figure 4c,g). Similarly, PEG 400 was selected as a humectant in the system according to the same reasons mentioned above (Figure 4d,h). Some previous studies suggested that higher molecular weight of surfactant led to the increased particle size [38], but our findings noted the oppose results since Tween 20^®^ (MW = 1227.54 g/mol, hydrophilic lipophilic balance (HLB) = 16.7) gave the smallest internal droplet size, followed by Brij S10^®^ (MW = 1151.56 g/mol, HLB = 12.0) and Triton X-114^®^ (MW = 624 g/mol, HLB = 12.4), respectively [36]. Although Tween 20^®^ is a nonionic surfactant, it could generate minus value of zeta potential because of the adsorption of anionic species derived from the materials used to make the NEs, such as free fatty acids (COO^−^) in the oil phase [39,40]. Furthermore, the results were in a good agreement with previous studies reported that rosemary oil encapsulation containing Tween 20^®^ had a negative zeta potential (−19.9 ± 4.6 mV) [41] as well as an edible solid lipid nanoparticle containing Tween 20^®^, of which its zeta potential was −12.8 ± 1.4 mV [42]. Additionally, the zeta potential of stearic acid-based solid lipid nanoparticle had consistently negative zeta potential in the range of −11 to −17 mV without any correlations between the zeta potential values and the amount of Tween 20^®^ in the formulation [38,41,42,43]. Lastly, the most suitable NE (Formulation C11), composed of 15% *w/w* tea seed oil, 7.5% *w/w* Tween 20^®^, 7.5% *w/w* PEG 400, and 70% *w/w* DI water, had small internal droplet size (170.0 ± 0.6 nm), narrow PDI (0.143 ± 0.010), and a pronounced zeta potential value (−24.0 ± 0.6 mV).

#### 3.2.4. Stability of Nanodelivery Systems

**Figure 5:**
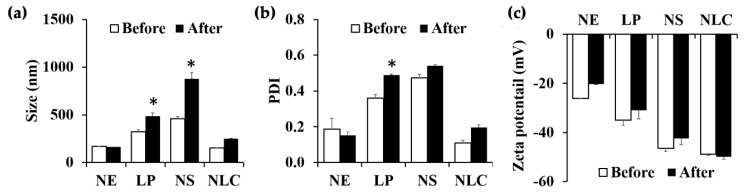
Stability of nanodelivery systems after heating–cooling cycles: (**a**) Internal droplet size; (**b**) PDI; and (**c**) zeta potential of nanoemulsion (NE), liposome (LP), niosome (NS), and nanostructured lipid carrier (NLC) before (□) and after (■) 8 cycles of heating–cooling cycles. Asterisk (*) denotes significantly different, *p* < 0.05.

The selected nanodelivery systems were investigated for their stability after storage in 8 cycles of heating–cooling conditions. NE and NLC still remained a homogeneous milky translucent solution, whereas aggregations of liposome and niosome particles were visually observed as the sediment at the bottom of the containers. The results as shown in Figure 5 also remarked that NE and NLC were stable since their particle size (Figure 5a), PDI (Figure 5b), and zeta potential (Figure 5c) remained unchanged after the stability test (*p* > 0.05). On the other hand, the particle size of liposome and niosome significantly increased (*p* < 0.05). It was remarked that nano-formulations with small internal droplet size and narrow PDI were more stable. Since narrow PDI represents the uniformity of internal droplet size and size distribution of the nanodelivery system, the narrow particle size distribution will typically result in more stable and elegant formulations [43,44]. Generally, zeta potential is a measure of the surface charge, which is important to control the particle interactions and to keep the internal droplets in a dispersed state [43,44]. Furthermore, the zeta potential greater than +30 mV or lower than −30 mV could be used to ensure the electrostatic stability since greater ionization at the interface tends to increase the electrostatic repulsion and to prevent the droplet aggregation [45,46]. Although liposome and niosome had lower zeta potential than −30 mV, they were not physically stable and the internal droplet size and PDI significantly increased after the heating-cooling cycles (*p* < 0.05). Therefore, NE and NLC were selected for further incorporation of the *O. sanctum* ethanolic extract.

### 3.3. Nanodelivery Systems Containing O. sanctum ethanolic extract

**Figure 6:**
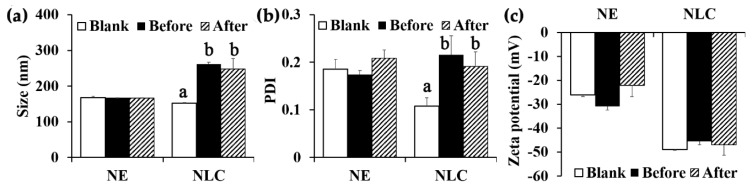
(**a**) Internal droplet size, (**b**) PDI, and (**c**) zeta potential of nanoemulsion (NE) and nanostructured lipid carrier (NLC) without *O. sanctum* ethanolic extract (□) and with *O. sanctum* ethanolic extract before (■) and after (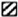) 8 cycles of heating–cooling stability test. The letters a and b denote values that were significantly different (*p* < 0.05).

NE and NLC containing 0.1% *w/w O. sanctum* ethanolic extract were homogeneous pale-dark green liquid. Internal droplet size, PDI, and zeta potential of both nanodelivery systems are shown in Figure 6. The internal droplet size of NLC containing *O. sanctum* ethanolic extract significantly increased from 152.5 ± 1.2 nm to 261.0 ± 5.3 nm (Figure 6a) and PDI significantly increased from 0.108 ± 0.18 to 0.216 ± 0.042 (Figure 6b), whereas those of NE remained the same after *O. sanctum* ethanolic extract incorporation (~168 nm). The likely explanation might be due to the swollen core of NLC loaded with *O. sanctum* ethanolic extract [47,48]. On the other hand, the zeta potential value of NLC was significantly more pronounced than that of NE. Their zeta potential values were −45.4 ± 2.4 mV and −30.9 ± 2.7 mV, respectively (Figure 6c). It was noted that the incorporation of *O. sanctum* ethanolic extract had no effect on the zeta potential value. Although NLCs had more pronounced zeta potential values than NEs, both formulations were stable. The droplet size, PDI, and zeta potential of *O. sanctum* ethanolic extract NE and NLC were not altered after heating–cooling conditions. Therefore, both nanodelivery systems were suitable for further use to deliver the *O. sanctum* ethanolic extract into dermal layer.

### 3.4. Irritation Study by Hen’s Egg Test on the Chorioallantoic Membrane (HET-CAM)

**Figure 7:**
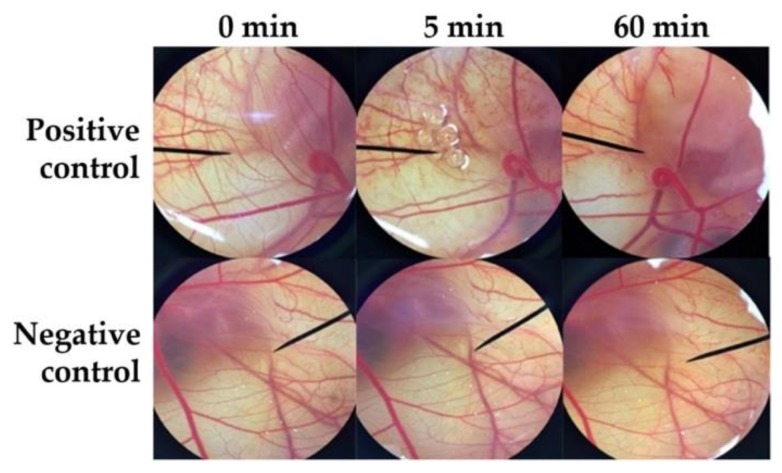
Photograph illustrating the effect of positive control (1% *w/v* SLS solution) and negative control (0.9% *w/v* NaCl) on chorioallantoic membrane before exposure the sample (0 min), after 5 min, and at the end of the experiment of 60 min.

**Figure 8:**
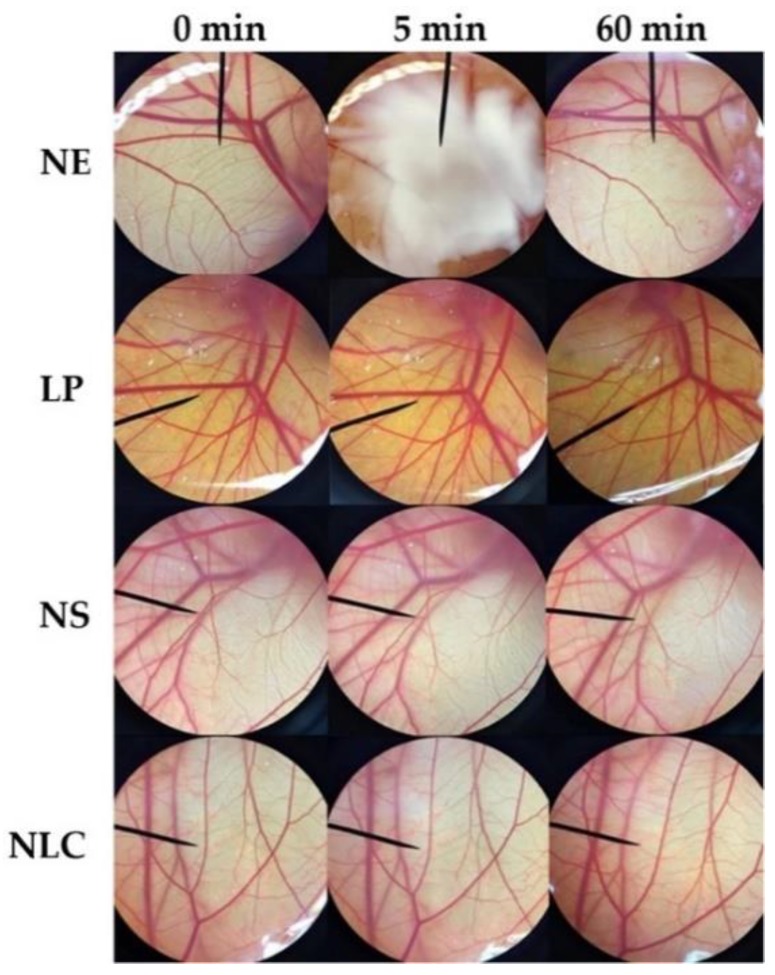
Photographs illustrating the effect of nanoemulsion (NE), liposome (LP), niosome (NS), and nanostructured lipid carriers (NLC) on chorioallantoic membrane before exposure the sample (0 min), after 5 min, and at the end of the experiment of 60 min.

Irritation is one of the major considerations for topical formulation development. The irritant effects can be observed in the CAM of fertile eggs following the exposure to a substance in the HET-CAM test which is analogous to the Draize rabbit-eye test [15]. The results as shown in Table 4 confirmed the reliability of the HET-CAM test on the irritation prediction since severe irritation was detected in a positive control and no irritation was detected in a negative control. Sodium lauryl sulphate (1% *w/w* SLS), a well-known irritant, induced severe irritation with the IS of 10.52 ± 0.8, whereas normal saline solution (0.9% *w/w* NSS), an isotonic aqueous solution, induced no irritation (IS = 0.0 ± 0.0). SLS solution immediately irritated the chorioallantoic membrane and generated hemorrhage, followed by coagulation and vascular lysis, respectively. All irritation signs were detected after 5 min of exposure, and they were more pronounced after 60 min, as shown in Figure 7. On the other hand, all dermal delivery systems had no effect on the CAM, as shown in Figure 8. Therefore, they were mild and safe nano-formulations for topical applications. However, liposome and niosome were not stable after 6 cycles of the heating–cooling stability test; they were hence not suggested for the further use and were omitted from further study.

Data are mean ± S.D (*n* = 3). Positive control was 1% *w/w* SLS, negative control was 0.9% *w/w* NSS, and 0.1% *w/w O. sanctum* solution was dissolved in 0.9% *w/w* NSS. N = no irritation and S = severe irritation. N.D. = not determined due to the instability of the formulation after 6 cycles of the heating–cooling stability test.

**Figure 9:**
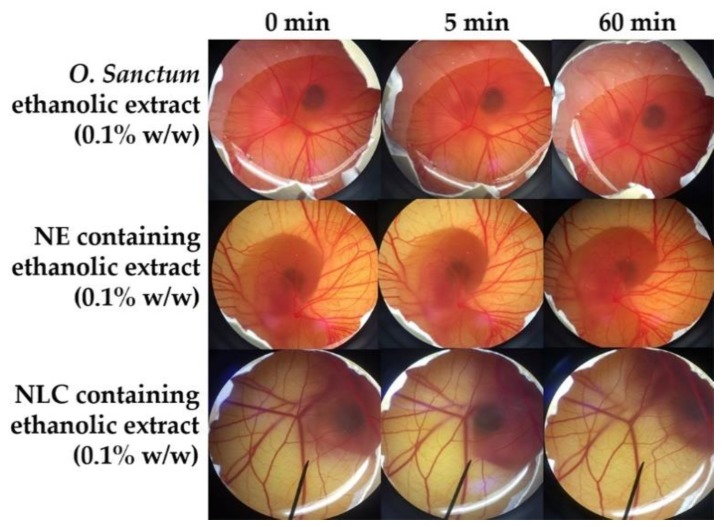
Photographs illustrating the effect of 0.1% *w/w O. sanctum* ethanolic extract, nanoemulsion (NE) containing 0.1% *w/w O. sanctum* ethanolic extract and nanostructured lipid carriers (NLC) containing 0.1% *w/w O. sanctum* ethanolic extract on chorioallantoic membrane before exposure the sample (0 min), after 5 min, and at the end of the experiment of 60 min.

The solution of *O. sanctum* ethanolic extract (0.1% *w/w* in 0.9% *w/w* NSS) induced no irritation in the HET-CAM test as shown in Figure 9. Additionally, both NLC and NE containing *O. sanctum* ethanolic extract also induced no irritation. Since the HET-CAM test has been suggested as a valid method reliably applied in a research on potentially toxic substances [49], it could be concluded that *O. sanctum* ethanolic extract both in the solution and nanodelivery system had no toxicity and was safe for topical applications.

### 3.5. In Vitro Release Profile of Dermal Delivery System Containing O. sanctum ethanolic extract

**Figure 10:**
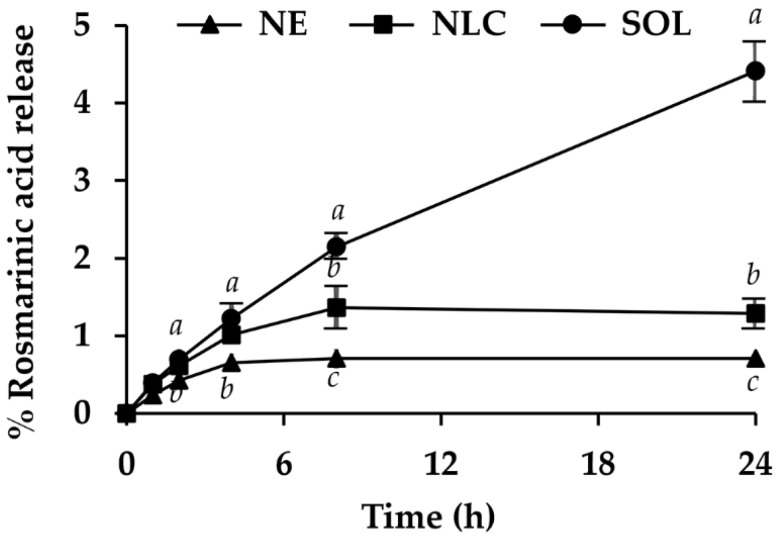
Rosmarinic acid (RA) release profile of nanoemulsion containing 0.1% *w/w O. sanctum* ethanolic extract (NE), nanostructured lipid carriers containing 0.1% *w/w O. sanctum* ethanolic extract (NLC), and 0.1% *w/w O. sanctum* ethanolic extract in propylene glycol (SOL): The letters a, b, and c denote significantly different content of rosmarinic acid (RA) released at each time interval (*p* < 0.05).

The release profile of RA, which was a major component of *O. sanctum* ethanolic extract, from NE and NLC are shown in Figure 10. *O. sanctum* ethanolic extract dissolved in propylene glycol was used as a control solution since the extract was not encapsulated in any nanodelivery system. The results noted that RA diffused from the solution to a receptor media in a zero-order manner with R^2^ of 0.974 (Table 5). Actually, the zero-order pattern of RA diffusion from the solution was obviously observed within 8 h (*R*^2^ = 0.992). The low diffused amount observed at 24 h might be due to the solubility limitation of RA in the media, which was 4% *w/w* BSA and 10% *w/w* Tween 80^®^ in PBS, pH 5.5.

According to the goodness-of-fit test, RA was released from NE and NLC in a Higuchi manner, especially within the first 8 h and tended to reach a steady state after that. NLC showed significantly higher release amounts of RA compared to NE (*p* < 0.05). However, their release amounts were significantly lower than that of the solution (*p* < 0.05). The release amounts of RA after 24 h from NE, NLC, and solution were 0.71 ± 0.05%, 1.29 ± 0.15%, and 4.41 ± 0.34%, respectively. According to the lower release amount and slower release rate of RA from NLC and NE compared to the solution, it could be remarked that both of them exhibited a controlled release pattern. The prolonged release of RA from NE was suggested to result from the rate-limiting step of RA diffusion across the interfacial film between the internal and external phases of NE [9,50], whereas the prolonged release of RA from NLC resulted from the Fickian diffusion of RA throughout the lipid matrix of NLC [51]. The release profiles of NLC can vary from very fast release, medium release, or extremely prolonged release depending on the matrix structure, the method of production, the solubilizing properties of the surfactant for the incorporated active, and the solubility of the active in the lipid matrix [52]. The present study remarked that 2.5% *w/w* Plantacare 2000^®^ could solubilize the *O. sanctum* ethanolic extract into the lipid matrix of cetyl palmitate and tea seed oil (5:1 by weight) very well and exhibited the control release pattern. The results were in a good agreement with the previous study of Aditya et al. [53] that reported that NLC and NE presented a pronounced quercetin release at the initial stage followed by a prolonged release. In addition, NLC revealed a faster release than NE, especially at the initial stage, but the cumulative quercetin amount released from NE was higher after 24 h [53].

### 3.6. In Vitro Skin Permeation and Skin Retention of Dermal Delivery System Containing O. sanctum Ethanolic Extract

**Figure 11:**
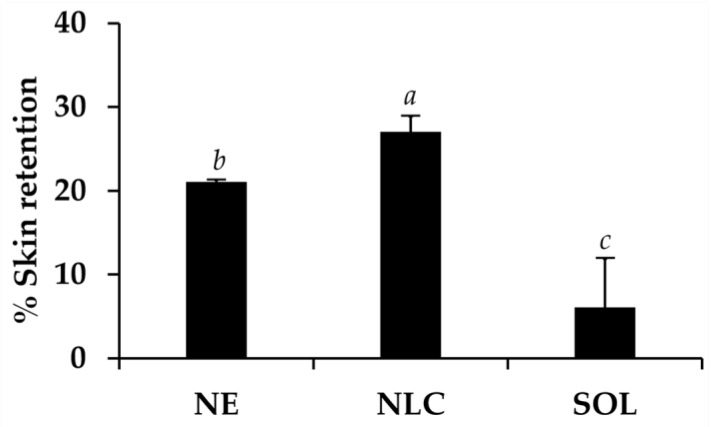
Skin retention of rosmarinic acid (RA) from nanoemulsion containing 0.1% *w/w O. sanctum* ethanolic extract (NE), nanostructured lipid carriers containing 0.1% *w/w O. sanctum* ethanolic extract (NLC), and so 0.1% *w/w O. sanctum* ethanolic extract in propylene glycol (SOL): The letters a, b, and c denote significantly different content of rosmarinic acid (RA) accumulated in the skin layer after 24 h exposure (*p* < 0.05).

The skin permeation of *O. sanctum* ethanolic extract in NE, NLC, and propylene glycol solution was investigated using Franz diffusion cell. None of the above formulations could deliver the extract thorough the piglet skin. Therefore, no systemic side effects occurred after topical application of these formulations. Skin retention amount of RA from NE, NLC, and propylene glycol solution is shown in Figure 11. NLC was the best formulation that could deliver the significantly highest amount of *O. sanctum* ethanolic extract into the skin layer (27.1 ± 1.8%), followed by NE (21.0 ± 0.3%) and propylene glycol solution (6.1 ± 5.8%), respectively (*p* < 0.05). The reason might be due to more lipophobicity of NLC than NE system. Since lipophilicity delivery systems were known to be compatible well with the skin fats both found in intercellular space of stratum corneum and intracellular space in the corneocyte, NLC could be more accumulated in those skin fats and led to higher skin retention content of the RA. Therefore, NLC was more appropriated for dermal delivery of RA, which possessed anti-ageing activity. The penetration to only a limited degree was desired to ensure that RA created only dermal effects and did not lead to pharmaceutical or systemic effects [54]. Furthermore, penetration of RA into each skin layer was suggested for further study to ensure whether RA accumulated in the stratum corneum, viable epidermis, or dermis layer.

## 4. Conclusions

RA, which has been reported as an anti-ageing compound, was a major component of *O. sanctum* ethanolic extract containing 19.3% *w/w*. However, RA was poorly absorbed into the skin since the skin retention amount of RA from *O. sanctum* ethanolic extract solution was only 6.1 ± 5.8%. NLC, which was a stable delivery system for *O. sanctum* ethanolic extract incorporation, and enhanced the dermal delivery of RA with the significantly higher skin retention amount of 27.1 ± 1.8% (*p* < 0.05). The suggested NLC formulation is composed of 0.1% *w/w O. sanctum* ethanolic extract, 5% *w/w* cetyl palmitate, 1% *w/w* tea seed oil, 2.5% *w/w* Plantacare 2000^®^, and 91.4% *w/w* DI water. It had an internal droplet size of 261.0 ± 5.3 nm, PDI of 0.216 ± 0.042, and zeta potential of −45.4 ± 2.4 mV. Additionally, this formulation showed a sustained release pattern with the RA release amount of 1.29 ± 0.15% after 24 h. Since no RA was detected in the receptor chamber of Franz cell in the skin permeation study, NLC was suggested as a delivery system specific for the dermal delivery. Furthermore, NLC containing *O. sanctum* ethanolic extract induced no irritation in HET-CAM test. Therefore, NLC was an attractive dermal delivery system that was safe and enhances dermal delivery of RA.

## Figures and Tables

**Table 1:** Composition of Nanostructured Lipid Carrier (NLC) formulations.

Ingredients	Concentration (% *w/w*)								
	A1	A2	A3	A4	A5	A6	A7	A8	A9
Stearic acid (SA)	5	-	-	-	-	-	-	-	-
Glyceryl monostearate (GMS)	-	5	-	-	-	-	-	-	-
Cetyl palmitate (CP)	-	-	5	3	1	5	5	5	5
Tea seed oil (TO)	1	1	1	1	1	2	3	3	3
Tween 20^®^ (T20)	2.5	2.5	2.5	2.5	2.5	2.5	2.5	-	-
Tween 80^®^ (T80)	-	-	-	-	-	-	-	2.5	-
Plantacare 2000^®^ (P2000)	-	-	-	-	-	-	-	-	2.5
DI water q.s.	100	100	100	100	100	100	100	100	100

**Table 2:** Composition of liposome and niosome formulations.

Ingredients	Concentration (% *w/w*)							
	B1	B2	B3	B4	B5	B6	B7	B8
Lecithin	1	3	5	3	3	-	-	-
Span 20^®^	-	-	-	5	10	5	5	5
Cholesterol	1	1	1	1	1	5	10	15
DI water q.s.	100	100	100	100	100	100	100	100

**Table 3:** Composition of nanoemulsion (NE) formulations.

Ingredients	Concentration (% *w/w*)										
	C1	C2	C3	C4	C5	C6	C7	C8	C9	C10	C11
Tea seed oil (TO)	5	-	-	10	15	20	35	15	15	15	15
Avocado oil (AD)	-	5	-	-	-	-	-	-	-	-	-
Almond oil (AL)	-	-	5	-	-	-	-	-	-	-	-
Tween 20^®^ (T20)	7.5	7.5	7.5	7.5	7.5	7.5	7.5	-	-	7.5	7.5
Brij S10^®^ (BS10)	-	-	-	-	-	-	-	7.5	-	-	-
Triton X-114^®^ (TX)	-	-	-	-	-	-	-	-	-	-	-
propylene glycol (PG)	7.5	7.5	7.5	7.5	7.5	7.5	7.5	7.5	7.5	-	-
Glycerin (Gly)	-	-	-	-	-	-	-	-	-	7.5	-
PEG-400 (PEG)	-	-	-	-	-	-	-	-	-	-	7.5
DI water q.s.	100	100	100	100	100	100	100	100	100	100	100

**Table 4:** Classification of dermal delivery systems with and without *O. sanctum* ethanolic extract according to the score of severity.

**Samples**	**Irritation Score**	
1% *w/w* SLS ^1^	10.52 ± 0.8 (S)	
0.9% *w/w* NSS ^2^	0.0 ± 0.0 (N)	
0.1% *w/w O. sanctum* solution	0.0 ± 0.0 (N)	
**Dermal Delivery Systems**	**Without Extract**	**With Extract**
Nanoemulsion	0.0 ± 0.0 (N)	0.0 ± 0.0 (N)
Liposome	0.0 ± 0.0 (N)	N.D.
Niosome	0.0 ± 0.0 (N)	N.D.
Nanostructured lipid carrier	0.0 ± 0.0 (N)	0.0 ± 0.0 (N)

^1^ SLS: sodium lauryl sulfate; ^2^ NSS: normal saline solution.

**Table 5:** Adjustment of the mathematical model to the release profile of *O. sanctum* ethanolic extract in nanoemulsion (NE), nanostructured lipid carriers (NLC), and solution (SOL).

Mathematical Model	Equation	Adjusted *R*^2^					
		NE		NLC		SOL	
		8 h	24 h	8 h	24 h	8 h	24 h
Higuchi	*Y* = *K* × *t*^0.5^	**0.946**	**0.716**	**0.988**	**0.770**	0.946	0.973
Zero order	*Y* = *K* × *t*	0.782	0.431	0.920	0.501	**0.992**	**0.974**
First order	ln *Y* = *K* × *t*	0.690	0.358	0.857	0.434	0.918	0.799

Bold values were the highest adjusted R-square of each column, NE = nanoemulsion containing *O. sanctum* ethanolic extract, NLC = nanostructure lipid carrier containing *O. sanctum* ethanolic extract, and SOL = solution of *O. sanctum* ethanolic extract in propylene glycol.

## References

[1] D’Haese P.S., Van Rompaey V., De Bodt M., Van de Heyning P. (2019). Severe Hearing Loss in the Ageing Population poses a Global Public Health Challenge. How can we better realise the Benefits of Cochlear Implantation to Mitigate this Crisis?. Public Health Front..

[2] Chávez L.A.C., García-Barrientos R., Ortega L.E.G., Garcia O.D., Alvarado M.I.E. (2019). Natural vs Synthetic Colors. In Anthocyanins-Novel Antioxidants in Human Health and Diseases Prevention. IntechOpen.

[3] Chaiyana W., Anuchapreeda S., Punyoyai C., Neimkhum W., Lee K.H., Lin W.C., Viernstien H., Mueller M. (2019). *Ocimum sanctum* Linn. as a natural source of skin anti-ageing compounds. Ind. Crops Prod..

[4] Roberts M.S., Mohammed Y., Pastore M.N., Namjoshi S., Yousef S., Alinaghi A., Haridass I.N., Abd E., Leite-Silva V.R., Benson H.A.E. (2017). Topical and cutaneous delivery using nanosystems. J. Control. Release..

[5] Ashtiani H.R.A., Bishe P., Lashgari N.A., Nilforoushzadeh M.A., Zare S. (2016). Liposomes in cosmetics. J. Skin Stem Cell..

[6] Ganesan P., Choi D.K. (2016). Current application of phytocompound-based nanocosmeceuticals for beauty and skin therapy. Int. J. Nanomed..

[7] Bnyan R., Khan I., Ehtezazi T., Saleem I., Gordon S., O’Neill F., Roberts M. (2018). Surfactant effects on lipid-based vesicles properties. J. Pharm. Sci..

[8] Gupta A., Eral H.B., Hatton T.A., Doyle P.S. (2016). Nanoemulsions: Formation, properties and applications. Soft Matter.

[9] Shaker D.S., Ishak R.A., Ghoneim A., Elhuoni M.A. (2019). Nanoemulsion: A Review on Mechanisms for the Transdermal Delivery of Hydrophobic and Hydrophilic Drugs. Sci. Pharm..

[10] Müller R.H., Mäder K., Lippacher A., Jenning V. (2000). Solid-liquid (semi-solid) liquid particles and method of producing highly concentrated lipid particle dispersions. German Patent Appl..

[11] Costa R., Santos L. (2017). Delivery systems for cosmetics-From manufacturing to the skin of natural antioxidants. Powder Technol..

[12] Müller R.H., Radtke M.S., Wissing A. (2002). Solid lipid nanoparticles (SLN) and nanostructured lipid carriers (NLC) in cosmetic and dermatological preparations. Adv. Drug Deliv. Rev..

[13] Deamer D., Bangham A.D. (1976). Large volume liposomes by an ether vaporization method. Biochim. Biophys. Acta Nucleic Acids Protein Struct..

[14] Schultz S., Wagner G., Urban K., Ulrich J. (2004). High-pressure homogenization as a process for emulsion formation. Chem. Eng. Technol..

[15] Luepke N.P., Kemper F.H. (1986). The HET-CAM test: An alternative to the Draize eye test. Food Chem. Toxicol..

[16] Steiling W., Bracher M., Courtellemont P., De Silva O. (1999). The HET–CAM, a useful in vitro assay for assessing the eye irritation properties of cosmetic formulations and ingredients. Toxicol. In Vitro.

[17] Somwongin S., Chantawannakul P., Chaiyana W. (2018). Antioxidant activity and irritation property of venoms from *Apis* species. Toxicon.

[18] Chaiyana W., Punyoyai C., Somwongin S., Leelapornpisid P., Ingkaninan K., Waranuch N., Srivilai J., Thitipramote N., Wisuitiprot W., Schuster R. (2017). Inhibition of 5α-reductase, IL-6 secretion, and oxidation process of *Equisetum debile* Roxb. ex vaucher extract as functional food and nutraceuticals ingredients. Nutrients.

[19] Chaiyana W., Phongpradist R., Leelapornpisid P., Anuchapreeda S. (2015). Microemulsion-based hydrogel for topical delivery of indomethacin. Int. J. Pharm. Pharm. Sci..

[20] Chaiyana W., Leelapornpisid P., Jakmunee J., Korsamphan C. (2018). Antioxidant and moisturizing effect of *Camellia assamica* seed oil and its development into microemulsion. Cosmetics.

[21] Laothaweerungsawat N., Neimkhum W., Anuchapreeda S., Sirithunyalug J., Chaiyana W. (2020). Transdermal delivery enhancement of carvacrol from *Origanum vulgare* L. essential oil by microemulsion. Int. J. Pharm..

[22] Ferderber K., Hook S., Rades T. (2009). Phosphatidyl choline-based colloidal systems for dermal and transdermal drug delivery. J. Liposome Res..

[23] Chaiyana W., Rades T., Okonogi S. (2013). Characterization and in vitro permeation study of microemulsions and liquid crystalline systems containing the anticholinesterase alkaloidal extract from *Tabernaemontana divaricata*. Int. J. Pharm..

[24] Sundaram R.S., Ramanathan M., Rajesh R., Satheesh B., Saravanan D. (2012). LC-MS quantification of rosmarinic acid and ursolic acid in the *Ocimum sanctum* Linn. leaf extract (Holy basil, Tulsi). J. Liq. Chromatogr. Relat. Technol..

[25] Kelm M.A., Nair M.G., Strasburg G.M., DeWitt D.L. (2000). Antioxidant and cyclooxygenase inhibitory phenolic compounds from *Ocimum sanctum* Linn. Phytomedicine.

[26] Håkansson A. (2015). Droplet breakup in high-pressure homogenizers. Engineering Aspects of Food Emulsification and Homogenization.

[27] Teeranachaideekul V., Souto E.B., Junyaprasert V.B., Müller R.H. (2007). Cetyl palmitate-based NLC for topical delivery of Coenzyme Q10–Development, physicochemical characterization and in vitro release studies. Eur. J. Pharm. Biopharm..

[28] Pathak P., Nagarsenker M. (2009). Formulation and evaluation of lidocaine lipid nanosystems for dermal delivery. AAPS Pharm Sci. Tech..

[29] Hu X., Zhang Y., Yang J., Wan H. (2014). Influence of liquid lipid content on the properties of puerarin-loaded lipid nanoparticles. J. Chin. Adv. Mater. Soc..

[30] Lasoń E., Sikora E., Ogonowski J. (2013). Influence of process parameters on properties of Nanostructured Lipid Carriers (NLC) formulation. Acta Biochim. Pol..

[31] Imran M., Revol-Junelles A.M., Paris C., Guedon E., Linder M., Desobry S. (2015). Liposomal nanodelivery systems using soy and marine lecithin to encapsulate food biopreservative nisin. LWT-Food Sci. Technol..

[32] Du M., Ouyang Y., Meng F., Zhang X., Ma Q., Zhuang Y., Liu H., Pang M., Cai T., Cai Y. (2019). Polymer-lipid hybrid nanoparticles: A novel drug delivery system for enhancing the activity of Psoralen against breast cancer. Int. J. Pharm..

[33] Briuglia M.L., Rotella C., McFarlane A., Lamprou D.A. (2015). Influence of cholesterol on liposome stability and on in vitro drug release. Drug Deliv. Transl. Res..

[34] Chandu V.P., Arunachalam A., Jeganath S., Yamini K., Tharangini K., Chaitanya G. (2012). Niosomes: A novel drug delivery system. Int. J. Novel Trends Pharm. Sci..

[35] Yadav J.D., Kulkarni P.R., Vaidya K.A., Shelke G.T. (2011). Niosomes: A review. J. Pharm. Res..

[36] Wang X.N., Chen Y.Z., Wu L.Q., Liu R.K., Yang X.H., Wang R., Yu K.W. (2008). Oil content and fatty acid composition of *Camellia oleifera* seed. J. Cent. South Univ. Forestry Technol..

[37] Quast K. (2016). The use of zeta potential to investigate the pKa of saturated fatty acids. Adv. Powder Technol..

[38] Shah R., Eldridge D., Palombo E., Harding I. (2014). Optimisation and Stability Assessment of Solid Lipid Nanoparticles using Particle Size and Zeta Potential. J. Phys. Sci..

[39] Teo A., Goh K.K., Wen J., Oey I., Ko S., Kwak H.S., Lee S.J. (2016). Physicochemical properties of whey protein, lactoferrin and Tween 20 stabilised nanoemulsions: Effect of temperature, pH and salt. Food Chem..

[40] Jo Y.J., Kwon Y.J. (2014). Characterization of β-carotene nanoemulsions prepared by microfluidization technique. Food Sci. Biotechnol..

[41] Ephrem E., Greige-Gerges H., Fessi H., Charcosset C. (2014). Optimisation of rosemary oil encapsulation in polycaprolactone and scale-up of the process. J. Microencapsul..

[42] Milsmann J., Oehlke K., Schrader K., Greiner R., Steffen-Heins A. (2018). Fate of edible solid lipid nanoparticles (SLN) in surfactant stabilized o/w emulsions. Part 1: Interplay of SLN and oil droplets. Colloids Surf. A Physicochem. Eng. Asp..

[43] Poluri K., Sistla R., Veerareddy P., Narasu L., Raje A., Hebsiba S. (2011). Formulation, characterization and pharmacokinetic studies of carvedilol nanoemulsions. Curr. Trends Biotechnol. Pharm..

[44] Fairhurst D., Dukhin A., Klein K. (2002). A new way to characterize stability and performance of cosmetic emulsions and suspensions. J. Cos. Sci..

[45] Clogston J.D., Patri A.K. (2011). Zeta potential measurement. Characterization of Nanoparticles Intended for Drug Delivery.

[46] Fang J.Y., Fang C.L., Liu C.H., Su Y.H. (2008). Lipid nanoparticles as vehicles for topical psoralen delivery: Solid lipid nanoparticles (SLN) versus nanostructured lipid carriers (NLC). Eur. J. Pharm. Biopharm..

[47] Tran T.H., Ramasamy T., Truong D.H., Choi H.G., Yong C.S., Kim J.O. (2014). Preparation and characterization of fenofibrate-loaded nanostructured lipid carriers for oral bioavailability enhancement. AAPS PharmSciTech..

[48] Bahari L.A.S., Hamishehkar H. (2016). The impact of variables on particle size of solid lipid nanoparticles and nanostructured lipid carriers; a comparative literature review. Adv. Pharm. Bull..

[49] Cazedey E.C.L., Carvalho F.C., Fiorentino F.A.M., Gremião M.P.D., Salgado H.R.N. (2009). Corrositex^®^, BCOP and HET-CAM as alternative methods to animal experimentation. Braz. J. Pharm. Sci..

[50] Morais J.M., Burgess D.J. (2014). In vitro release testing methods for vitamin E nanoemulsions. Int. J. Pharm..

[51] Meng F., Asghar S., Gao S., Su Z., Song J., Huo M., Meng W., Ping Q., Xiao Y. (2015). A novel LDL-mimic nanocarrier for the targeted delivery of curcumin into the brain to treat Alzheimer’s disease. Colloids Surf. B Biointerfaces.

[52] Souto E.B., Müller R.H. (2008). Cosmetic features and applications of lipid nanoparticles (SLN^®^, NLC^®^). Int. J. Cosmet. Sci..

[53] Aditya N.P., Macedo A.S., Doktorovova S., Souto E.B., Kim S., Chang P.S., Ko S. (2014). Development and evaluation of lipid nanocarriers for quercetin delivery: A comparative study of solid lipid nanoparticles (SLN), nanostructured lipid carriers (NLC), and lipid nanoemulsions (LNE). LWT-Food Sci. Technol..

[54] Mu R.H. (2005). Solid lipid nanoparticles (SLN) and nanostructured lipid carriers (NLC) for dermal delivery. Percutaneous Absorption.

